# What happens when the rain is back? A hypothetical model on how germination and post-germination occur in a species from transient seed banks

**DOI:** 10.1371/journal.pone.0229215

**Published:** 2020-02-26

**Authors:** Bruna Luiza de Souza, João Paulo Ribeiro-Oliveira, Juliana Pereira Bravo, Gabriela Fernanda Dias, Edvaldo Aparecido Amaral da Silva

**Affiliations:** 1 Departamento de Produção e Melhoramento Vegetal, Faculdade de Ciências Agronômicas, Universidade Estadual Paulista, Botucatu, São Paulo, Brazil; 2 Instituto de Ciências Agrárias, Universidade Federal de Uberlândia, Uberlândia, Minas Gerais, Brazil; Brigham Young University, UNITED STATES

## Abstract

We hypothesize that by simulating the natural priming in seeds of a species that forms transient seed banks it is possible to clarify molecular aspects of germination that lead to the recruitment of seedlings when the next rainy season begins. We used seeds of *Solanum lycocarpum* as a biological model. Our findings support the idea that the increment of seed germination kinetics when the rainy season returns is mainly based on the metabolism and embryonic growth, and that the hydropriming, at the end of seed dispersion, increases the germination window time of these seeds by mainly increasing the degradation of galactomannan of the cell wall. This can improve the energy supply (based on carbon metabolism) for seedling growth in post-germination, which improves the seedling’s survival chances. From these findings, we promote a hypothetical model about how the priming at the end of the rainy season acts on mRNA synthesis in the germination of seeds from transient banks and the consequence of this priming at the beginning of the following rainy season. This model predicts that besides the Gibberellin and Abscisic Acid balance (content and sensitivity), Auxin would be a key component for the seed-seedling transition in Neotropical areas.

Seed collection was performed under authorization number SISGEN AB0EB45.

## Introduction

The formation of soil seed banks is what gives dynamism to natural forests and thus guarantees the flow of life on the planet. Guarded by the dormancy state and/or ability to interrupt metabolism when there is water scarcity, these seeds remain in anhydrobiosis in the soil for brief periods (seeds of species that form transient banks) or long periods (seeds of species that form permanent banks)[1,2]. This is a species-specific ecophysiological aspect based on the genetic and/or epigenetic record that includes material from several specimens that have established themselves in an area [3–6]. Thus, when we understand how the soil seed bank works, we do not only outline strategies for in situ conservationism of species, but also ex situ (germplasm banks). In this context, several reports have brought light to the functionality of these banks, relying mainly on longevity and the germination-dormancy balance of seeds [e.g., 7 and their references]. However, we still know little about what affects this germination-dormancy balance from a molecular point of view. A strategy to fill this gap is to cross classic physiological measurements of the germination ecophysiology with key components of the gene expression involved in embryo growth [8].

A limiting factor to perform a crosstalk between gene expression and germination ecophysiology would be that laboratory protocols are not very representative of what happens in the field from an ecological point of view. However, this barrier is being overcome by the idea that detailed laboratory protocols for sampling communities and performing germination experiments can produce significant characters that are as enlightening as those obtained in field experiments. Therefore, if we have these protocols, we will be able advance the understanding of functional plant traits [see 9]. For example, the interruption of seed germination *sensu stricto* can be considered natural in environments in which the seasonal rains do not always coincide with the best environmental perspective (temperature and humidity of the substrate and the environment) for the establishment of a young plant [10,11], as is the case in Neotropical areas. As a consequence, the embryo interrupts the intra-seminal development, resuming it only at the beginning of the next rainy season. In this new season, germination will occur in a shorter time and the seedling will have greater environmental resources for its initial establishment [2]. This context describes a form of natural physiological priming of seeds, but the scientific community has not explored this in the context of the molecular seed biology of native species. This is due to the difficulties of experimental standardization in the field regarding factors that interrupt the germination process. A solution could be to simulate the field in the laboratory, casting light on an important question: How does germination and post-germination occur in seeds that form transient banks when the new rainy season starts?

To answer the question, we can use the seed priming technique. This technique dates back to the 70s and was developed primarily to improve uniformity of initial plant growth of cultivated species with asynchronous establishment [12]. The principle is the same as natural priming, based on the interruption of the germination *sensu stricto* [13,14]. The practicality of the technique and the intriguing aspects of the seed-seedling transition process, made it also very opportune for pioneer studies on water and osmotic stresses [12,14]. What we gain in using this technique, in this case, is understanding, under laboratory conditions, how different constraints of the soil water potential promote responses on a physiological phenomenon [see 15]. After all, as soil drainage is gradual, the reduction of soil water potential tends to be cadenced over days or months [16]. This justifies the various studies considering the reduction of water potential, mainly through the osmotic increment in the substrate. However, these studies do not explain which aspects are involved in the resumption of embryo growth when the new rainy season starts. Taking this into account, hydropriming, one of the most popular seed priming techniques, can help us understand how the seed, more specifically the embryo, modulates the development due to changes in a seasonal environment, such as in Neotropical areas. Hydropriming promotes a gradual reduction of available water to seeds without affecting the osmotic potential of the substrate [14]. After this treatment, the seeds are stored to be used whenever they are needed. This scenario is closer to what is observed in Neotropical areas without problems of soil salinity, such as the Cerrado [see 16].

The idea that we can understand the seed-environment relationship through embryonic development comes from the fact that the embryo is the main germination modulator. As a consequence, the expression of genes associated with key enzymes becomes one of the main molecular components we have to elucidate [2,8]. Among the genes expressed, some have robust validation, not only for model species, but also for congeners, such as: *LeEXP8*, *LeEXP10* (Expansins) [17], expressed in the embryo; LexG1 [Polygalacturonase (EC 3.2.1.15)] [18], expressed at the apex of the embryo radicle and at the micropylar region; *LeMside1* [(Endo-β-mannanase; EC 3.2.1.78)] [19], *LeMan2* [(β-mannosidase; EC 3.2.1.25)] [20] and *LeaGal* [(α-galactosidase; EC 3.2.1.22)] [21], expressed in the micropylar region. Taking this into account, our hypothesis is that by simulating the natural priming in seeds of a species that forms transient seed banks, we can clarify molecular aspects of germination that lead to the recruitment of seedlings at the beginning of the new rainy season. If we can validate this hypothesis, our findings will promote insights on the interruption of germination *sensu stricto* of native species of a seasonal environment. Thus, our expectation is not only to promote basic knowledge about the mechanisms involved in the seed-seedling transition from an ecophysiological point of view, but also to give clues about which studied genes can be used as markers of the germinative state of a native species that are used in ecological restoration projects. Our aim is to promote a bridge between basic and applied knowledge, elaborating a hypothetical model of seed germination metabolism after natural priming promoted by late rains in a seasonal environment.

## Material and methods

### Biological model

*Solanum lycocarpum* A. St. -Hil. forms transient seed banks and has a zoochorous seed dispersal system (see dispersion system in [22]), but its seeds have no dormancy type. Here, it is important to note that we previously considered seeds of this species as dormant, since the last germination was around 40 days after sowing [23]. However, recently, all research concerning seed germination of the species demonstrates that the last germination of seed samples from different numbers of mother plants occurs around 10–15 days, reaching high germinability (70–90%) [8,24]. Taking this into account, the species was considered a biological model for molecular studies on seed germination of non-dormant species from Neotropical areas [8]. The species is an arborescent plant typical of the Cerrado (a biodiversity hotspot), and therefore has undergone a selective pressure that has forced native populations to specialize in particular environments because of high biome fragmentation [25,26]. Besides having this positive impact on the flora, this species also has repercussions on the fauna [see 25]. Additionally, the species is considered a nurse plant (*sense* [27]), and therefore promotes facilitation in ‘stressful environments’ [28], improving the success rate of restoration of degraded environments [29]. However, local communities have been exploiting it for some time, since it possesses potential for the fruit industry [30].

### Fruit origin and seed sample processing

We collected ripe fruits of *Solanum lycocarpum* directly from the mother plants (*n* = 20) established around Lavras, MG, Brazil [21° 14’ 43”S, 44° 14’ 43’'W; at 919 meters above mean sea level (MAMSL)]. The mother plants were established at least 20 m from each other in natural vegetation, and the choice of these plants depended on the number of fertile individuals and the quality and number of fruits produced. We opened the fruits and removed the pulp and passed it through a sieve under flowing water to separate the seeds. We only used visually similar seeds in relation to morphology. Therefore, we excluded apparently damaged, empty and immature seeds from the sample. The seeds in hygroscopic equilibrium had a water content of 10% (35% RH; 25 °C). We determined this water content by the oven method at 105 °C for 17 hours [31].

### Seed priming

We placed the seeds in hygroscopic equilibrium in tubes containing 15 mL of water and kept them at 15 °C for 15 days, according to [24]. Subsequently, we placed the tubes in a shaker (Multifunctional mixer MR-II, Biomixer, São Paulo SP, Brazil) throughout the incubation period. We dried the seeds in a controlled environment in a Biochemical Oxygen Demand germination chamber or B.O.D. (B.O.D., Technal, São Paulo SP, Brazil) with 35% RH at 25 °C using closed boxes containing a saturated solution of MgCl_2_ solution. All this process occurred in 16 hours. After that, we stored the seeds at 20 °C in dry substrate until sowing.

### Seed germination analyses

We performed the germination test in a B.O.D., alternating temperatures at 20 and 30 °C, and using a photoperiod of 12 hours (12 light/12 dark) under white fluorescent light (15.35 ± 2.54 μmol m^2^ s^-1^ Photosynthetic Photon Flux Density–PPFD). We used a sample (*n*) with 200 seeds, which was segregated into eight replicates (*r*) [sub-samples (*ss*) or plot size (*ps*)] with 25 seeds each (*n* = 200; *r* = 4; *ss* or *ps* = 50). We performed a completely randomized design (CRD). We sowed the seeds on germination paper in germination boxes. We dampened the paper with distilled water with a volume equivalent to 2.5 times the dry mass of the paper in milliliters. We maintained paper moisture throughout the test by adding 1 mL of distilled water whenever necessary.

We carried out the germination evaluations every 24 hours, and the germination criterion was the radicle protrusion. We terminated the analyses 15 days after sowing when the remaining seeds presented coalescing tissues (i.e., advanced decomposition process). We analyzed primed and non-primed seeds.

Besides germinability [G (%); estimates from regression curve from accumulative germination over time] and Maguire’s rate [*Rate* (a frequentist daily germination measurement); embryo protrusion day^-1^] [32], we calculated the absolute germination measurements, which are: time to first (*t*_f_; days) and last germination (*t*_l_; days), mean germination time ($\overset{-}{t};$ days), mean germination rate ($\overset{-}{v}$; days^-1^), uncertainty (*U*; bits) [33], coefficient of variation of the germination time (CV_t_; %) and synchronization index (*Z*) [34]. We also calculated the range from time to first and last germination in the sample (λ = range of the seed germination time) [8]. We plotted graphs showing the relative frequencies of seed germination [35].

### Gene expression analysis

We extracted the RNA from two sources: 50 embryos and micropylar endosperms subjected to priming, and 50 embryos and micropylar endosperms imbibed in water. We used the NucleoSpin RNA Plant^®^ kit (Macherey-Nagel, Bethlehem PA, USA) to perform the RNA extraction from both groups. We froze all of this material in liquid nitrogen and ground it down to a powder with a mortar and pestle. We added approximately 100 mg of the ground powder to 350 μL of the extraction buffer (RA1) and 3.5 μL of β-mercaptoethanol (β-ME) and then homogenized it by vortexing. We transferred these samples to the NucleoSpin Filter^®^ and centrifuged (~13 000 G-Force). We quantified the extracted RNA using a Nanodrop-2000 spectrophotometer (Thermo Scientific, Wilmington DE, USA) and confirmed RNA integrity in a 1% agarose gel.

For cDNA preparation, we used the First-Strand cDNA Synthesis Kit (New England Biolabs, Ipswich MA, USA). We performed the cDNA synthesis from RNA of primed and non-primed seeds at 1, 5 and 10 days after imbibition. These times correspond to a predominantly biophysical phase, a predominantly biochemical phase and the end of the germination *sensu stricto* (embryo protrusion) of *Solanum lycocarpum* seeds, respectively [8].

For each biological sample, we used 1 000 ng of total RNA (RT) + 0.8 μL of 25X dNTP Mix (100 mM) + 2 μL 10X RT of random primers + 2 μL of 10x RT Buffer + 1 μL MultiScribeTM Reverse Transcriptase and 4.2 μL Nuclease-free H_2_O. We incubated the samples at 70 °C for 5 min and then dipped them in ice. We placed the samples in a thermocycler at a temperature of 25 °C for 10 min, at 37 °C for 2 h and at 85 °C for 5 min for inactivation of the enzymes. We quantified the cDNA in a Nanodrop-2000 spectrophotometer (Thermo Scientific, Wilmington DE, USA).

We studied the following genes: expansin, α-galactosidase and polygalacturonase in the embryo, and α-galactosidase, β-mannosidase, endo-β-mannanase and polygalacturonase in the micropylar endosperm (S1 Table). We used the sequences deposited in the NCBI database (http://www.ncbi.nlm.nih.gov/) for tomato (*Solanum lycopersicum* L.) for the primer design. For the development of primers, we used the Primer Quest software (https://www.idtdna.com/primerquest/Home/Index) (S2 Table).

We used an Eco Real-Time (Illumina) with primers, cDNA and Eva-Green_ master mix to perform Real-Time PCR. For each reaction, we used 3 μL of cDNA (60 ng L^-1^), 0.25 μL of forward and reverse primers (at a concentration of 10.0 mM) and 5 μL of Master Mix Eva-Green for a final volume of 10.0 μL sample^-1^. The amplification protocol consisted of 2 min at 50 °C, 5 min at 95 °C; then 40 cycles of 15 s at 95 °C and 1 min at 60 °C ± 1°C.

We determined melting curves to assess specificity. We used the universal 18S primers as an internal control to normalize the products. We performed real-time PCR three times using biological replicates. We calculated values of fold change in gene expression in relation to 1 day of imbibition, using 2^-ΔΔCt^.

### Statistical analysis

For the classical physiological measurements recorded from CRD, we tested the data for the assumptions (α = 0.01) of normality of residuals (Kolmogorov-Smirnov test) and homogeneity of variances (Levene test), and, when the assumptions were violated, we performed data transformation according to [36] (S3 Table). For cumulative germination curves, we chose regression models based on significance from ANOVA test and model fitting to observed data.

For molecular raw data, we used the bootstrap method with 1 000 re-samplings, since values generated above this number are similar according to the convergence test. Bootstrap is a technique of successive resampling from original data [37], which ensures that analytical models are reproducible and reliable. The technique is useful in applications where analytical confidence intervals are unobtainable or when robust nonparametric confidence intervals are required [38]. In addition, the bootstrap easily estimates the distribution of an estimator, reduces impacts of outlier and numerical anomalies, as well as calculates the estimates of standard error and population parameters of confidence intervals [39]. For molecular comparisons of fold change, we used the tool REST^®^ for Ct calculation, which was based on the data simulation of bootstrap methods from raw data. The REST performs comparative quantifying from the Pair-Wise Fixed Reallocation Randomization Test method [40]using a normalizing gene. For clarification of the REST results, we used the overlapping of confidence intervals from bootstrap estimative, according to [8].

## Results

**Fig 1:**
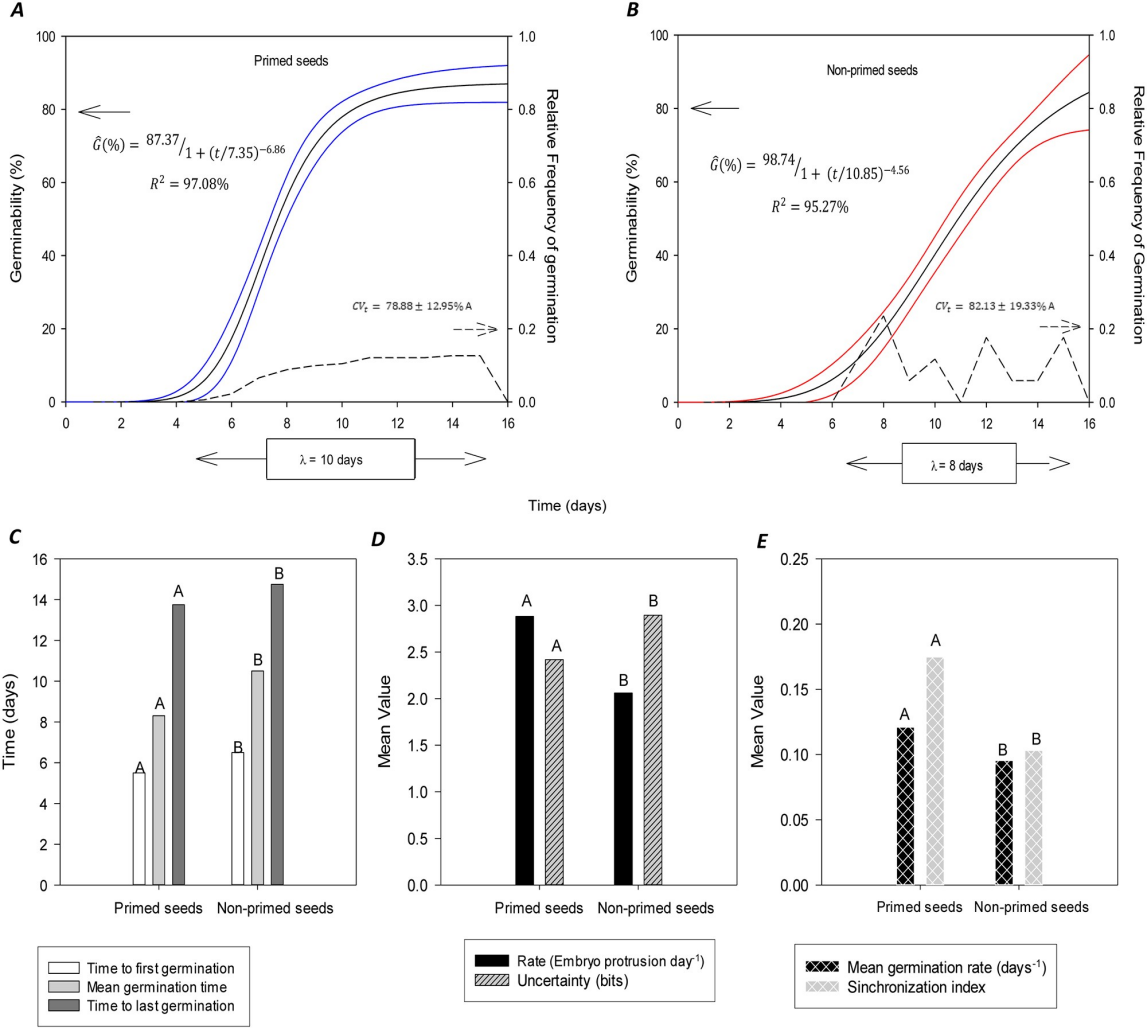
Germination process in primed and non-primed seeds (*n* = 200; *r* = 4; *ss* = 50) of *Solanum lycocarpum* A. St.-Hil. ***A***, ***B***: Cumulative curves of germinability (filled lines) and relative frequency of germination over experimental time (dashed lines). Black line: estimative of cumulative germinability curve; colored lines (Blue: primed seeds; Red: Non-primed seeds): upper and lower limits of confidence intervals from bootstrap method (1 000 resamples). $\hat{G}$: Germinability from Gompertz model; *R*^*2*^: Coefficient of determination of the germinability model (fitting germinability model). *CV*_t_: coefficient of variation of the germination time; λ: germination time range. ***C***, ***D*** and ***E***: Classical germination measurements: time [***C***. time to first (*t*_f_) and last germination (*t*_l_), and mean germination time ($\overset{-}{t}$)], frequentists (***D***. *Rate*), velocity [***E***. mean germination rate ($\overset{-}{v}$)] and sinchrony [***D***. Uncertainty (*U*); ***E***. Synchronization index (*Z*)]. In this case, averages followed by the same capital letter within the treatment (primed vs non-primed) do not differ by the Tukey test (α = 0.05).

The germinability of *Solanum lycocarpum* seeds primed by water is similar to that of non-primed seeds, fitting the Gompertz model with three parameters (see CI in germinability curves; Fig 1A and 1B). However, seed hydropriming leads to a smaller and more constant relative frequency of germination (Fig 1A and 1B), increasing the time window for potential germination of seed samples by up to two days (ʎ = 10 days). This time reduction in seed germination range is not associated with the increment of uniformity of the germination process (CV_t_; Fig 1A and 1B), but with the precocity of the first (t_f_) and last (t_l_) seed germination in the sample. These outliers were responsible for the anticipation of the germination peak of the seed sample ($\overset{-}{t}$) in approximately one day (Fig 1C). This relation between the germination frequency, the time window and the germination time makes the average frequency of embryo protrusions (*Rate*) higher in primed seeds (Fig 1D). As a consequence, there is a less predictable (*U*; Fig 1D) and more synchronous (Fig 1E) germination process (*Z*). This is because water priming increases germination metabolism ($\overset{-}{v}$)(Fig 1E), promoting a more active physiological process.

**Fig 2:**
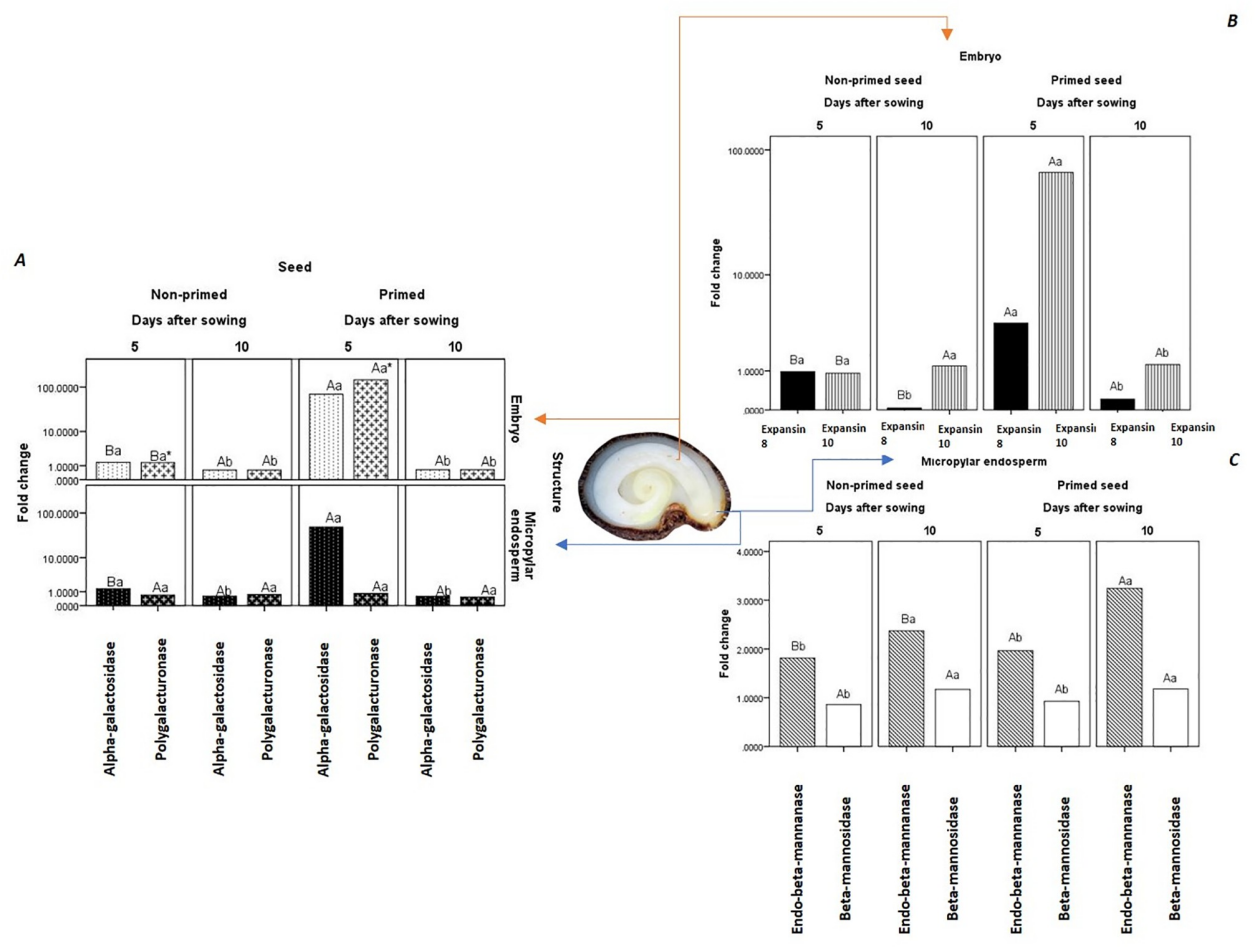
Relative fold change in transcript abundance of genes related to embryo and micropylar endosperm in germinating seeds of *Solanum lycocarpum* A. St.-Hil. imbibed in water. ***A***. mRNA fold change of α-galactosidase (*LeaGal*) and polygalacturonase (*LexG1*) expressed in the embryo and in the micropylar endosperm; ***B***. mRNA fold change of Expansin 8 (*LeEXP8*) and Expansin 10 (*LeEXP10*) expressed in the embryo; ***C***. mRNA fold change of endo-β-mannanase (*LeMside1*) and β-mannosidase (*LeMan2*) expressed in the micropylar endosperm. Averages followed by the same capital letter within the treatment (primed vs non-primed seeds) do not differ by the Pair-Wise Fixed Reallocation Randomization Test (α = 0.05); *: Differences between gene expression in embryo and micropylar endosperm indicated by the overlapping of interval confidence (α = 0.05) calculated from bootstrap method (1 000 resampling) applied to raw data. For each biological triplicate we used 50 embryos (*n* = 150; *r* = 3; *ss* = 50).

The increase of the germination metabolism in hydroprimed seeds can also be observed by increasing the gene expression of mRNAs coding key enzymes for embryo growth and/or weakening of the micropyle endosperm (Fig 2A, 2B and 2C). In general, only β-mannosidase (*LeMan2*) and polygalacturonase (PG; *LeXPG1*), expressed in the micropylar endosperm, are insensitive to seed hydropriming, regardless of the germination *sensu stricto* phase (Fig 2A and 2C). Another peculiarity of these enzymes is that β-mannosidase increases its expression in the micropylar region during germination *sensu stricto*, and PG maintains the same fold change of the predominantly biochemical phase (classically called Phase II, 5 DAS; Fig 2C). However, it is important to emphasize that the gene expression of PG in the embryo was higher than in the micropylar endosperm region during the predominantly biochemical germination phase (5 DAS), being substantially more expressed (approximately 100 times more) in primed seeds (Fig 2A).

α-galactosidase (*LeaGal*) was also substantially more expressed (up to 100 times more) in primed seeds during the predominantly biochemical phase, but had similar expression in both embryo and the micropylar endosperm regions (Fig 2A). What draws attention to these two enzymes is the fact that there is a drastic reduction in the expression in primed seeds at the end of germination, when there is embryo protrusion (10 DAS; Fig 2A and 2C). On the other hand, endo-β-mannanase (*LeMside1*) increases its expression over germination time, especially in primed seeds (Fig 2C). In this case, the highest expression of the gene codifying the enzyme occurs at the end of germination, but this expression is higher in hydroprimed seeds (72% higher than 5 DAS) in relation to those unprimed (60% higher than 5 DAS).

Similar to the gene expression of hydrolytic enzymes associated with catabolism of the cell wall, the expansins, in general, increased their expression in hydroprimed seeds (Fig 2B). The greatest impact of the technique on the mRNA of expansin isoenzymes (Expansin 8: *LeEXP8* and Expansin 10: *LeEXP10*) occurred at 5 DAS in primed seeds, where the expression of Expansin 10 was 100 times higher than in non-primed seeds. For non-primed seeds, the Expansin 10 gene had its peak expression at the end of germination, even though this value was not much higher than at 5 DAS. The values of Expansin 10 were the same at 10 DAS for both primed and non-primed seeds. In non-primed seeds, the peak expression of Expansin 8 was at 5 DAS being up to ten times higher than at 10 DAS. This expression pattern of Expansin 8 also occurred in hydroprimed seeds, but the variation of expression between 5 and 10 DAS was much higher (approximately seven times higher). These findings support the idea that the increment of germination velocity in *Solanum lycocarpum* seeds when a new rainy season starts is mainly based on embryo metabolism, and that the seed hydropriming, from late rains after seed dispersion, improves the germination time window of these seeds. This occurs because the hydropriming from late rains mainly increases the degradation of galactomannan of the cell wall, improving the energy supply for embryo growth.

## Discussion

By using *Solanum lycocarpum* as a biological model and seed hydropriming as an experimental technique to simulate the natural conditioning from rains at the end of the rainy season in tropical and seasonal environments, we demonstrate that: (i) non-dormant seeds of a species forming transient seed banks have similar germinability to the moment of post-dispersion, and (ii) these seeds have a greater time window to express germination than those dispersed at the beginning of the new rainy season. This is not related to the increment of germination uniformity, but with a small and constant frequency of daily germination, as pointed by relative frequency of germination and *Rate* measurement. Taking this into account, seed hydropriming from late rains seems to affect the seed physiology in a species forming transient seed banks in a different way than hydropriming affects seed physiology of a cultivated species [see 14; 12]. In cultivated species, seed hydropriming not only increases germination uniformity, but also germination velocity. In contrast, our findings infer that the physiological gain from rainfall priming is relative only to the germination kinetics. This was demonstrated by mean germination rate and ratified by the high fold change of mRNA codifying key enzymes for weakening the cell wall as well as the cell extension in seed structures over germination time.

At first, germination precocity could be attributed only to embryo growth *per se*, since it is conditioned to the perfect relation between embryo growth potential and micropylar endosperm degradation. These characteristics are related to mechanical resistance (higher or lower) to embryo protrusion [41]. However, this relation also reminds us of the idea of mobilizing reserves for embryo growth. This is because our model species possesses an endospermic seed, which is characterized by micropylar cell walls thickened by mannans [23]. The mannans have already been shown to be a fundamental energy supplier for early establishment (seed germination and seedling or young plant development) of native species [42,43]. This hypothesis is even clearer when taking into account that mRNAs codifying both β-mannosidase and polygalacturonase (PG) expressed in the micropylar region have the same pool between hydroprimed and non-primed seeds, but that α-galactosidase and PG are highly expressed in the embryo region during the predominantly biochemical germination phase. During embryo protrusion, these enzymes decay their gene expression, contrasting with the increment of the expression of endo-β-mannanase and β-mannosidase in the micropylar endosperm region. By acting on the mannans metabolism in the cell wall of the micropylar endosperm, these enzymes not only reduce the mechanical resistance of the region, but also release complexed carbohydrates that, when degraded by α-galactosidase, become readily useful for early seedling development [19,44–47]. This explains the high expression of the enzyme both in the embryo and in the micropylar region, making it the key to the seed-seedling transition for species forming a transient seed bank. This is an unprecedented component of the study of the germination of tropical native seeds and demonstrates that, in addition to the oxidative metabolism [8], it is also fundamental for seeds of species that form transient seed banks in tropical areas. Therefore, cell wall modifying enzymes are also fundamental to the success of the seed-seedling transition and can be a molecular marker to evaluate the quality of seed samples that will be used to recompose degraded areas. We recommend that gene expression related to endo-β-mannanase and α-galactosidase activity (in our case *LeMside1* and *LeaGal*, respectively) be considered in pretesting of potential seed samples to be used in recovery projects.

It is noteworthy that PG is involved in the development processes of conductive vessels in seedlings [see 48], causing its greater expression in the embryo. This is a healthy indicative of improvement in the survival of seedlings that emerged from seeds hydroprimed by rain in a previous rainy season. Another addendum that deserves attention is the similar gene expression of PG in the micropylar region of primed and non-primed seeds. Because it is associated with the metabolism of pectin residues, PG has been one of the most important components for biotic interactions between plants and the environment, conforming development processes to interactions favorable to the individual [49]. As it remains constant, even with seed hydropriming, the enzyme may play an important role in recognizing biotic interactions during the germination of seeds that form the transient bank. In this sense, there may be an important synergy with β-mannosidase, also insensitive to rehydration, but fundamental for the germination of endospermic seeds such as Solanaceae [see 2]. This idea should be better explored in future studies on the proteomic germination of seeds that form soil seed banks.

In addition to the high gene expression of PG, two other components draw our attention to the embryo: (i) the high expression of Expansin 10 in hydroprimed seeds during the predominantly biochemical germination phase, and (ii) maintenance of the gene expression pattern of Expansin 8 in the embryo of primed and non-primed seeds. A first explanation is that Expansin 10 is more expressed at the end of germination [see 17] and, when anhydrobiosis is resumed at the end of the rainy season, the pool of mRNA codifying expansins would be preserved. Consequently, it would increase not only the *de novo* synthesis of these enzymes when rehydrated, but also the activation of mRNA of the previous pool. This explanation would also be valid for the largest pool of most enzymes in hydroprimed seeds. However, Expansin 8 maintains a similar magnitude of increment expression between the predominantly biochemical germination phase and embryo protrusion in primed and non-primed seeds. Therefore, endospermic seeds that form transient seed banks can be particularly interesting biological models, since the greatest potential of embryo growth in hydroprimed seeds is mainly subsidized by Expansin 10, whose half-life seems to fall over storage time [17]. This explains the fact that embryo growth is potentially higher in hydroprimed seeds still in the predominantly biochemical phase and demonstrates why seeds that form transient seed banks have a higher survival rate of seedlings than those from the new season [e.g., 50].

**Fig 3:**
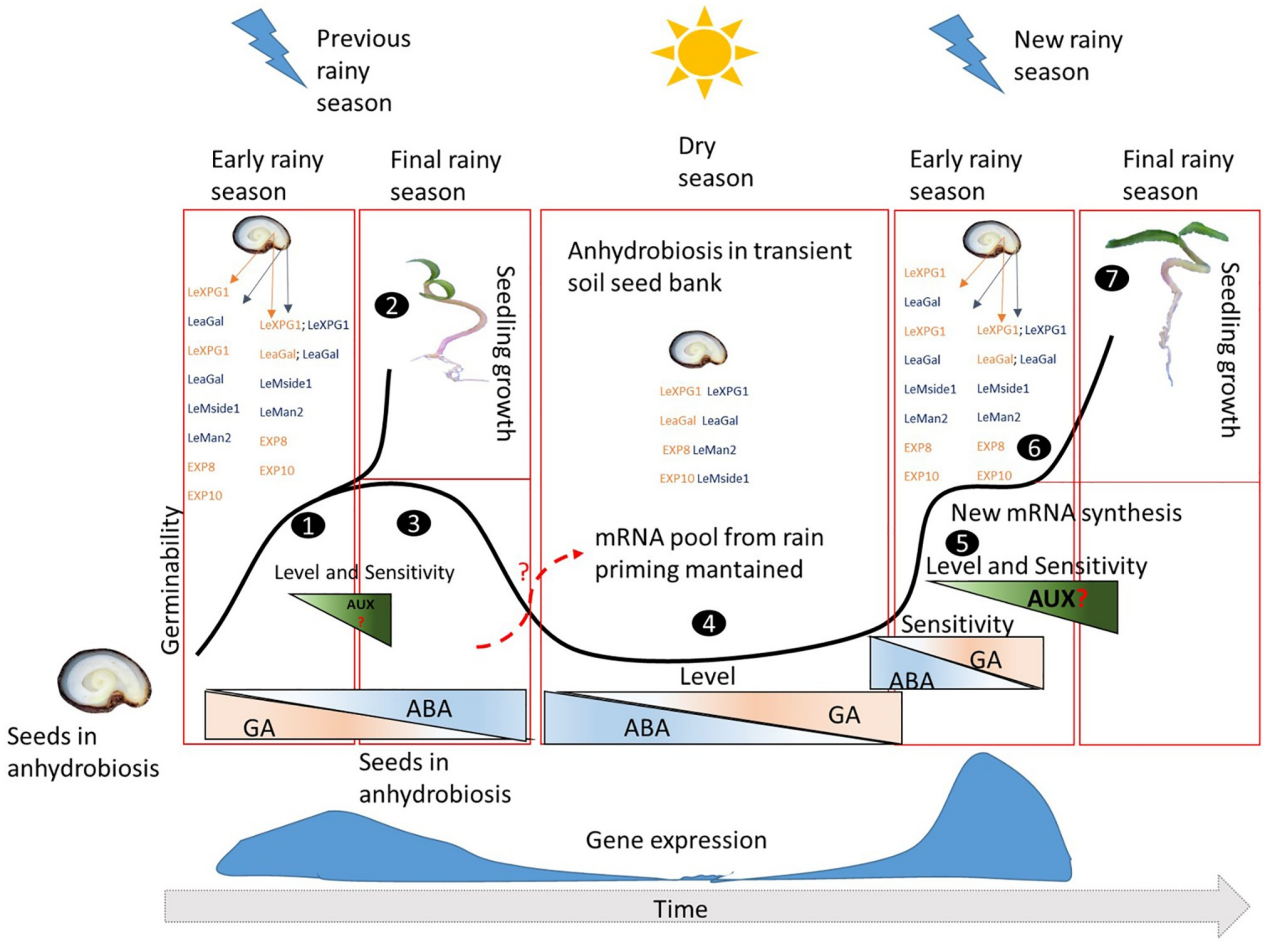
A hypothetical model of seed-seedling transition in a species forming transient seed banks without any type of dormancy in a tropical area. ***1***. Scattered seeds during the rainy season have high germinability, but the asynchronous and slower process causes only part of them to reach the end of germination. The majority of mRNA coding key enzymes for the degradation of the micropylar endosperm and/or expansion of the embryo cells increase the fold change until the moment of protrusion, highlighting the endo-β-mannanase (*LeMside1*) in the micropylar region and Expansin 10 (*LeEXP10*) in the embryo. ***2***. With water available, the seedlings that emerged from the seeds developed at the end of the previous rainy season show better growth, which may be associated with increased content and sensitivity to auxin. One point that reinforces this idea is the fact that both expansins (*LeEXP8* and *LeEXP10*) and polygalacturonases (*LeXPG1*), whose expression is usually associated with auxin, have a greater increment in fold change. ***3***. Delayed seeds present an increment in the synthesis and sensitivity to abscisic acid (ABA) in counterpoint to the reduction of the content and sensitivity to Gibberellin (GA). This probably is due to the gradual reduction of water availability. Thus, the synthesis of mRNA codifying hydrolases and Expansins, which are GA-regulated and ABA-repressed, falls dramatically. This causes the seeds to fail germinating even without presenting dormancy, but without major damage to germinability. These seeds enter the transient seed bank in the soil. ***4***. In the transient seed bank, anhydrous seeds probably maintain the pool of mRNA of hydrolytic enzymes (endo-β-mannanase, α-galactosidase–*LeaGal–*and β-mannosidase–*LeMan2*) and Expansins synthesized during the end of the rainy season. It is possible that this pool remains until the return of the rains due to the high content of ABA. ***5***. As the rains gradually return, not only do the GA levels increase, but also the sensitivity to the hormone. Thus, we have two simultaneous actions: the pool of mRNA codifying enzymes involved in embryo growth and/or the weakening of the micropylar endosperm is activated, and the synthesis of new enzymes is triggered. As a consequence, gene expression in general, is suddenly increased. In this case, embryo growth is rapidly triggered by means of polygalacturonases and expansins (8 and 10). This rapid activation and synthesis speeds up germination without influencing time uniformity, increasing the possibility of germination of seeds produced in the previous rainy season. ***6***. The greater expression of polygalacturonase and expansins already in the middle of the predominantly biochemical germination phase is probably due to a higher activity and content of auxin. This could explain greener seedlings with better development from seeds that were hydroprimed by the rains, explaining the general consensus that these seeds can promote seedlings with a higher probability of survival. ***7***. The seeds, hydroprimed at the end of the previous rainy season, are not only the fastest to germinate, but also the first to develop seedlings. The hydrolytic activity of the micropylar endosperm and the intense expansion activity of the embryo cells probably allow them to better use the rains of the new season. Thus, these genes can be molecular markers of reliable seed samples for environmental conservation/recovery programs of biodiversity hotspots, such as the Cerrado in the Neotropical area.

What is common to all enzymes that increase their expression when seeds are hydroprimed is the fact that they are GA-dependent and ABA-repressed [2]. This information encouraged us to release a hypothetical model (Fig 3) in which, at the end of the rainy season, seeds without dormancy in the transient seed bank regress to anhydrobiosis due to the increment of the ABA pool. ABA prevents enzymatic activity, but it will not act on the mRNA pool of GA-dependent enzymes synthesized at the end of the previous rainy season. When rainfall resumes, the increase in GA and the reduction of ABA levels causes the mRNA synthesized in the previous rainy season to be expressed, and enzyme synthesis to be activated again. The consequence is possibly a greater sensitivity to GA and, consequently, an increase in the velocity of metabolic reactions that culminate in the precocity of germination, which occurs at more constant rates, allowing seedlings to emerge more quickly and in a smaller time window. Another aspect that is probably involved in the germination of seeds forming transient banks is the content and sensitivity to auxin. Although few reports discuss the role of auxin in germination *sensu stricto* [see 51], the elevation of the expression of mRNA codifying polygalacturonase and expansins after hydropriming may be indicative that the hormone has a key role when the predominantly biochemical germination phase starts; in addition, since both enzymes are typically related to the hormone [52], greener and larger seedlings may be a direct consequence of the increment of hormone activity. Auxin is an important morphogen [53]and, therefore, may be associated with improved embryo sensitivity to the maternal environment, in order to allow for a faster seed-seedling transition for seeds scattered at the end of the previous rainy season. This model can also be used to explain the ‘hydration memory’ provoked by hydration cycles in the environment, as is the case with seeds from the Brazilian caatinga [see 54,55]. We would like to emphasize that this model is hypothetical, but it is a contribution for future papers concerning molecular aspects of early plant development from a transient seed bank. For these studies, our model can be a guide to a new experimental design based not only on indirect inferences of hormonal balance from gene expression, but also on a hormonal profile of AUX, GA and ABA, as well as on transcripts and enzymes specifically associated with each one. Thus, our model stimulates future work with a crosstalk between germination and post-germination ecophysiology and proteomic germination.

## Conclusions

We understand that rain priming at the end of a rainy season in *Solanum lycocarpum* seeds, a species forming transient seed banks in the soil, improves seed germination kinetics when the rains return, which leads to an increased expression of genes associated with embryo growth and with enzymes involved in the degradation of the micropylar endosperm. Thus, gene expression studies on seed priming may contribute to the development of molecular markers to assess the way that rain priming and or artificial seed priming techniques improve seed quality and hence decrease the time for seedling production for species from areas targeted for natural recovery, such as Cerrado (a hotspot in biodiversity) in Neotropical areas.

## Supporting information

S1 Table Studied genes (mRNAs) in the embryo and the micropylar endosperm during germination of *Solanum lycocarpum* seeds. (DOCX)

S2 Table Specific oligonucleotides to the embryo and micropylar endosperm regions of *Solanum lycocarpum* seeds used for real-time PCR analysis. (DOCX)

S3 Table Values of statistics and probability (*P*) of classical germination measurements of *Solanum lycocarpum* A. St.-Hil. seeds. (DOCX)
