# Supplementary material for: What happens when the rain is back? A hypothetical model on how germination and post-germination occur in a species from transient seed banks
Source: PLoS One. 2020 Feb 26;15(2):e0229215. doi: 10.1371/journal.pone.0229215 (PMC7043802; doi:10.1371/journal.pone.0229215)
Supplement: S1 Table — (DOCX) [file pone.0229215.s001.docx]

**S1 Table.** Studied genes (mRNAs) in the embryo and the micropylar endosperm during germination of *Solanum lycocarpum* seeds.

| **Gene symbol** | **Gene name** | **Function** |
| --- | --- | --- |
| ***LeMan2*** | Endo-β-mannanase | Hydrolysis of cell-wall polysaccharides ^1^ |
| ***LeMside1*** | β-mannosidase | Hydrolysis of cell-wall polysaccharides ^2^ |
| **LeaGal** | α-galactosidase | Hydrolysis of cell-wall polysaccharides ^3^ |
| **EXP8** | Expansin 8 | Weakens the non-covalent binding between cell-wall polysaccharides (Extension) ^4^ |
| **EXP10** | Expansin 10 | Weakens the non-covalent binding between cell-wall polysaccharides (Extension) ^4^ |
| ***LeXPG1*** | Polygalacturonase | Reduces the molecular size of pectic polymers by cleavage of side-chain residues ^5^ |
| **18S** | 18S ribosomal | Normalizer^6^ |

^1^ Nonogaki et al. (2000); ^2^Mo et al. (2002); ^3^Feurtado et al. (2001); ^4^Chen et al. (2001); ^5^Sitrit et al. (1999);

^6^ kim et al. (2003)
