# Supplementary material for: What happens when the rain is back? A hypothetical model on how germination and post-germination occur in a species from transient seed banks
Source: PLoS One. 2020 Feb 26;15(2):e0229215. doi: 10.1371/journal.pone.0229215 (PMC7043802; doi:10.1371/journal.pone.0229215)
Supplement: S2 Table — (DOCX) [file pone.0229215.s002.docx]

**S2 Table.** Specific oligonucleotides to the embryo and micropylar endosperm regions of *Solanum lycocarpum* seeds used for real-time PCR analysis.

| **Name** | **Sense** | **Antisense** |
| --- | --- | --- |
| **Endo-β-mannanase^1^** | 5’ CTCGCGATCAAGCTGACTATT3’ | 5’CAATAGAGACTCGCAGGGAAATA3’ |
| **β-mannosidase^1^** | 5’GGATTGTCCCTGTTGCTTACA3’ | 5’GACGTCTGGGATGACTTGAATC3’ |
| **α-galactosidase^1^** | 5’GATTCCGCTACTGAGCCTTATATT3’ | 5’GATCACAATGATGGACCACAAAC3’ |
| **Expasin 8^1^** | 5’CCCTCCATTCCAACCTCTAAC3’ | 5’CTGAGTCCCAGCATCAGAATAA3’ |
| **Expansin 10^1^** | 5’1GCCAAACTTCCCTTTCTGTTTC3’ | 5’CCGCCATCTCCATCCTAAAT3’ |
| **Polygalacturonase** | 5’CGTTCGTCACCAGCACTAAA3’ | 5’TCCTGGCCCCATACCATCTATAA3’ |
| **18S ribosomal^2^** | 5’TGACGGAGAATTAGGGTTCG3’ | 5’CCTCCAATGATCCTCGTTA3’ |

^1^ Designed by the author; ^2^ Kim et al. (2003)
