# Supplementary material for: What happens when the rain is back? A hypothetical model on how germination and post-germination occur in a species from transient seed banks
Source: PLoS One. 2020 Feb 26;15(2):e0229215. doi: 10.1371/journal.pone.0229215 (PMC7043802; doi:10.1371/journal.pone.0229215)
Supplement: S3 Table — (DOCX) [file pone.0229215.s003.docx]

**S3 Table.** Values of statistics and probability (*P*) of classical germination measurements of *Solanum lycocarpum* A. St.-Hil. seeds.

| **Statistics** | | | **Characters** | | | | | | | | | | | | | | | | | |  |
| --- | --- | --- | --- | --- | --- | --- | --- | --- | --- | --- | --- | --- | --- | --- | --- | --- | --- | --- | --- | --- | --- |
|  |  |  | ***t_f_* (day)** | | | $\bar{\boldsymbol{t}}$ **(day)** | | ***tl (day)*** | | ***CV_t_* (%)** | | ***U* (bit)** | | ***Z*** | | ***Rate***  ***(*embryo protrusion day^-1^)** | | | $\bar{\boldsymbol{v}}$ **(day^-1^)** | | |
| **ANOVA Assumptions** | | ***W***  **(*P*)** | | 0.83  (0.01) | | 0.83  (0.02) | | 0.74  (0.01) | | 0.82  (0.01) | | 0.93  (0.24) | | 0.89  (0.06) | | 0.87  (0.03) | | | 0.83  (0.01) | | |
|  | | **^1^*F***  **(*P*)** | | 7.00  (0.02) | | 0.71  (0.41) | | 3.73  (0.07) | | 1.37  (0.26) | | 0.25  (0.62) | | 0.16  (0.70) | | 0.14  (0.71) | | | 7.88  (0.07) | | |
| **Model** | **Source of Variation** | **DF** | **MS** | | **^2^*F***  **(*P*)** | **MS** | **^2^*F***  **(*P*)** | **MS** | **^2^*F***  **(*P*)** | **MS** | **^2^*F***  **(*P*)** | **MS** | **^2^*F***  **(*P*)** | **MS** | **^2^*F***  **(*P*)** | **MS** | **^3^*F***  **(*P*)** | **MS** | | **^2^*F***  **(*P*)** |  |
| **One-Way ANOVA** | Primed *vs* Non-primed seed | 1 | 4.00 | | 4.67  (0.04) | 19.31 | 80.52 (0.00) | 0.07 | 5.01  (0.04) | 110.55 | 0.48  (0.50) | 0.91 | 17.87 (0.00) | 0.02 | 30.56 (0.00) | 2.70 | 18.40  (0.00) | 0.003 | | 72.14  (0.00) |  |
|  | Error | 14 | 0.86 | | - | 0.24 | - | 0.01 | - | 232.04 | - | 0.05 | - | 0.0007 | - | 0.15 | - | 0.00004 | | - |  |
|  |  | ***CV* (%)** | 15.43 | |  | 5.21 |  | 3.20 |  | 19.16 |  | 8.49 |  | 18.66 | - | 15.50 |  | 5.57 | |  |  |

*W*: statistic of Shapiro-Wilk test for residual normality (*P* ≥ 0.01); ^1^*F*: statistic of Levene test for homogeneity of variances (*P*≥ 0.01); DF: Degrees of Freedom; MS: Mean Square; ^2^*F*: statistic of Snedecor test (One-Way ANOVA; *P* < 0.05); *t_f_*_: time to first germination;_$\bar{t}$: mean germination time; *t_l_*; time to last germination; *CV_t_*: coefficient of variation of the germination time; *U*: Uncertainty of germination process; *Z*: synchronization index; *Rate*: Maguire’s rate ;$\bar{v}$: mean germination rate;. The statistical analyses for characters were based on transformed data by √x or, in case of percentage data, by arcsine $\sqrt{x/{100}}$. C.V.: Coefficient of Variation.
